# IDSL.GOA: Gene Ontology Analysis for Metabolomics

**DOI:** 10.1101/2023.03.25.534225

**Published:** 2023-07-03

**Authors:** Priyanka Mahajan, Oliver Fiehn, Dinesh Barupal

**Affiliations:** 1 Integrated Data Science Laboratory for Metabolomics and Exposomics, Department of Environmental Medicine and Public Health, Icahn School of Medicine at Mount Sinai, New York, USA 10954; 2 NIH-West Coast Metabolomics Center, University of California, Davis, California, 95616, USA

**Keywords:** Gene ontology, metabolomics, over-representation, enrichment analysis, pathway analysis, Cytoscape, metabolic networks, aging, Expasy, KEGG, NCBI

## Abstract

Biological interpretation of metabolomics datasets often ends at a pathway analysis step to find the over-represented metabolic pathways in the list of statistically significant metabolites. However, definitions of biochemical pathways and metabolite coverage vary among different curated databases, leading to inaccurate and contradicting interpretations. For the lists of gene, transcripts and proteins, Gene Ontology (GO) terms over-presentation analysis has become a standardized approach for the biological interpretation. But, GO analysis has not been achieved for metabolomics datasets. We present a new knowledgebase and the online tool, Gene Ontology Analysis by the Integrated Data Science Laboratory for Metabolomics and Exposomics (IDSL.GOA) to conduct GO over-representation analysis for a metabolite list. The IDSL.GOA knowledgebase covers 2,324 metabolic GO terms and associated 2,818 genes, 22,264 transcripts, 20,158 proteins, 1,482 EC annotations, 2,430 reactions and 2,212 metabolites. IDSL.GOA analysis of a case study of older vs young female brain cortex metabolome highlighted over 250 GO terms being significantly overrepresented (FDR <0.05). On contrast, for the same metabolite list, MetaboAnalyst and Reactome Pathway Analysis suggested less than 5 pathways at FDR <0.05. We showed how IDSL.GOA identified key and relevant GO metabolic processes that were not mentioned by alternative pathway analysis approaches. Overall, we suggest that metabolomics researchers should not limit the interpretation of metabolite lists to only pathway maps and can also leverage GO terms as well. IDSL.GOA provides a powerful tool for this purpose, allowing for a more comprehensive and accurate analysis of metabolite pathway data. IDSL.GOA tool can be accessed at https://goa.idsl.me/

## Introduction:

Metabolism is a fundamental biological process of living organisms that transfers energy and matter among them, supporting adaptations and essential biological processes from the cell-cycle to reproduction. About 17% of annotated genes and their products (transcripts and proteins) in the human genome are involved in regulating and catalyzing metabolic processes through enzymatic transformations and transport mechanisms ^1^. These metabolic genes function in a highly coordinate fashion to operate a metabolic network of linked reactions that provide energy, substrates, signaling and defense metabolites, as well as neutralizing foreign harmful chemicals^1^. Many metabolic processes such a glycolysis or purine biosynthesis are conserved across all domains of life^2^ and several such a steroid biosynthesis are specific to mammalians. Analysis of these metabolic genes, their products, and endogenous metabolites using omics approaches, including genomics, transcriptomics, proteomics, and metabolomics, can identify metabolic processes that are important during different life stages in normal and adverse health conditions^3^. These molecular omics approaches let us discover new insights into how metabolic processes are altered by diseases, toxic exposures and baseline genetic variations, enabling new prevention and therapeutic strategies for human diseases^4–7^.

Metabolomics enables the simultaneous study of multiple metabolic processes, including pathways, transport, and reactions. Metabolomics assays are diverse and complex in terms of their analytical conditions, but they can generate quantitative and semi-quantitative data for hundreds of endogenous metabolites^8^. Recently reported datasets can have between 1,500 to 2,000 named metabolites and several thousand unidentified metabolites^8, 9^. These metabolites originate from overlapping pathways of catabolic and anabolic reactions and can also be biomarkers for metabolic processes^10^. Environmental, genetic, or biological factors can alter the regulatory, signaling, and enzyme kinetic mechanisms in one or more metabolic pathways and processes, leading to altered levels of related metabolites in cells, tissues or body fluids^11, 12^. For example, aging reprograms carbohydrate and lipid metabolism pathways in the liver^13^, tobacco smoke exposure alters the nucleotide and reactive oxidative stress species metabolism^14^, and FADS gene polymorphisms alter the levels of circulating PUFAs^15^. We can expect to see a continuous growth in the number of named metabolites in metabolomics datasets due to new advances^16, 17^ in analytical techniques and computational methods and resources.

One of the key challenges in utilizing metabolomics datasets is how to interpret these large chemical lists for mechanistic insights^18^. The first step in interpreting metabolomics datasets is to pre-process the data to remove any noise or artifacts that may affect the accuracy of the analysis^19^. This typically involves normalization, scaling, and filtering of the data. Once the data has been pre-processed, it can be analyzed using a variety of statistical and bioinformatics tools, including univariate and multivariate analysis, pathway and network analysis, and machine learning algorithms^18^. Pathway and network analysis can provide mechanistic insights into the biological pathways linked to the altered metabolites^20^. To gain a better understanding of the biological mechanisms underlying the metabolic alterations, metabolomics data can be integrated with other types of data, such as transcriptomics, proteomics, and genomics data^3^. This can provide a more comprehensive view of the underlying biological processes and their interactions. Interestingly, metabolomics datasets often have metabolites that are yet to be connected to a biochemical reaction and pathway^21, 22^. To also include these poorly studied metabolites, hybrid approaches of the atomic mapping of reaction and chemical similarity network (MetaMapp) and enrichment analysis (ChemRICH) can be used^21, 22^. A manual curation process to link metabolites in metabolomics datasets to biomedical literature is inefficient to cover the ever-growing volume of the literature. Therefore, an automated process to create the chemical to publication linking can be used to identify the prior publication that can support the mechanistic interpretation obtained from the pathway and network analysis^23^. Finally, it is important to validate the mechanistic interpretation using independent datasets or follow up experiments^7^ to provide further support for the underlying biological mechanisms.

To interpret a list of significant metabolites, protein or genes, in the context of functional and biological relationships among them, a pathway analysis approach is often used to find the pathways that are significantly over-represented in the input list^10^. A hypergeometric test is typically conducted for a background database dependent pathway analysis^18^. The background pathway information for these approaches can be obtained from Kyoto Encyclopedia of Genes and Genomes(KEGG)^24^, BioCyc^25^ and Reactome^26^, which are representative and curated biochemical databases^18^. In parallel with these pathway analyses, gene and protein lists are also often interpreted using gene ontology (GO) term enrichment analysis^27^, which covers terms that relate to pathways as well as other biological processes such as cell cycle or apoptosis, or even pathways that are not yet included in other biochemical databases. A GO term analysis can provide a comprehensive interpretation of an input list of genes, proteins, and metabolites. However, there is not yet a single tool developed that can perform a GO analysis for a metabolite list.

We have developed a new tool named ‘IDSL.GOA’ (Gene Ontology Analysis by the Integrated Data Science Laboratory for Metabolomics and Exposomics) to perform GO enrichment analysis for a list of metabolites. The tool is supported by a knowledge base representing a metabolic network consisting of genes, nucleotides, proteins, enzymes, reactions, and reactants (metabolites) that are directly sources from National Center for Biotechnology Information (NCBI), KEGG, Expasy and GO consortium databases. We present a case study of an aging mouse metabolic atlas to highlight the metabolic processes that were suggested to be related to the aging process and were only identified by the IDSL.GOA based GO analysis method. The online tool is available at https://goa.idsl.me/ site.

## Material and methods:

### IDSL.GOA Knowledgebase:

We assembled and integrated information from a diverse set of data sources, including genes, transcripts, proteins, enzymes, reactions, compounds, atomic-pairs, gene ontology terms and the relationships among them. Table 1 provides the web addresses for the publicly available data sources and their respective locations. To focus specifically on metabolism, we restricted our gene selection to those related to GO term GO:0008152 (metabolic process) and linked with the human genome. Only the downstream entities for these metabolic genes were included in the knowledgebase. Utilized identifiers for creating the knowledgebase were - NCBI Gene, NCBI Protein, NCBI Nucleotide, GO Term, Enzyme Commission Number (EC), KEGG reaction and KEGG compound. Linkages among these entities were extracted or accessed from the resources listed in Table 1.

### Over-representation statistics:

For the GO analysis, we employed an overrepresentation analysis (ORA) test using the hypergeometric distribution. This statistical test is a widely accepted method for determining whether a set of molecular entities (gene or proteins or metabolites) is significantly overrepresented in a particular biological pathway or process, given a background database. We also applied filters 1) overlap >= 3, 2) at least three genes in the GO process and 3) the overlap should be >5% of the total set size for a GO term. The overlap represents how many out of the input KEGG list are found among the KEGG identifiers linked with a GO term. These filters narrow down the list of GO terms to only the most relevant ones, ensuring that our analysis was focused and accurate for the relationships between metabolites and GO terms.

We have used “phyper” function in R to compute the hypergeometric test. The parameter for the test were – *phyper(x-1,y,a,b, lower.tail = FALSE)*, where x is the overlap between the input list of KEGG identifiers and compounds linked with a GO term, *y* is the count of all compounds linked with the GO term, *a* is the count of all compounds not linked with the GO-term (2,212-y), *b* is the count of the KEGG identifiers from the input list that were found in the KB. By default, the *phyper* function in R calculates the probability of drawing less than or equal to *x* for a GO term. Use of the parameters “x-1” and “lower.tail=FALSE” returns the probability of drawing more than or equal to *x* for a GO term. The total number of KEGG identifiers linked with GO terms was 2,212. For example, for the Nucleoside salvage (GO:0043174), *x* was 13, *y* was 61, *b* was 62, and *a* was 2150 (2,212-62) for the test study’s results. The *p*-value of this GO term was computed as ‘*phyper(12,61,2150,62, lower.tail = FALSE)’* which returns 4.218424e-09.

The IDSL.GOA tool uses the False Discovery Rate (FDR) cutoff of 0.05 to control the proportion of false positives in multiple hypothesis testing in GO analysis. We repeated this test for all metabolically relevant 2,324 GO-terms.

### Case study and its analysis.

Our test study was based on publicly available data from the Aging Mouse Brain Metabolome Atlas^8^, a comprehensive resource that provides information on the metabolites found in the different regions of brain of aging mice. Specifically, we compared the brain metabolome of the cortex region in an older female mouse against that of a young mouse. To identify the significantly different metabolites, we used the student t-test. To perform IDSL.GOA overrepresentation analysis, we needed to map the PubChem identifiers to KEGG identifiers. We obtained the necessary mapping information from the PubChem Identifier Exchange Service ( https://pubchem.ncbi.nlm.nih.gov/idexchange/idexchange.cgi ), which provides a convenient web interface for converting between various types of chemical identifiers. Using this tool, we converted the PubChem identifiers for the compounds in our test study to their corresponding KEGG identifiers which were used as input for IDSL.GOA analysis. Specifically, we used KEGG identifiers for the compounds that had a *p*-value of less than 0.05 in the student t-test. The same KEGG identifier list was used as input for a pathway analysis by Reactome^26^ and MetaboAnalyst^28^ tools.

### IDSL.GOA online tool:

The online tool was developed using the ReactJS JavaScript framework (https://reactjs.org/), which is known for its efficient rendering of dynamic user interfaces. To facilitate data visualization, we utilized the Google Chart (https://developers.google.com/chart ) and Cytoscape JS plugins (https://github.com/plotly/react-cytoscapejs ), specifically designed to work with ReactJS. Google Chart enabled us to create interactive charts, while Cytoscape JS was instrumental in generating network diagrams that depicted the relationships among genes, transcripts, proteins, enzymes, reactions, compounds, and atomic-pairs. By leveraging these informatics tools, we were able to provide users with a seamless experience for analyzing metabolite lists. Cytoscape online version is a lightweight and user-friendly tool that allows users to perform basic network visualization and analysis tasks without the need to install the software locally. For small networks, the online version may be sufficient, but for larger and complex network, it is recommended to download the Cytoscape SIF (Simple Interaction Format) file and use the local version of Cytoscape software to create high resolution graphics. Instructions to use the IDSL.GOA tool are provided on the landing page.

## Results:

### Creating the IDSL.GOA metabolic knowledgebase (KB):

**Figure 1:**
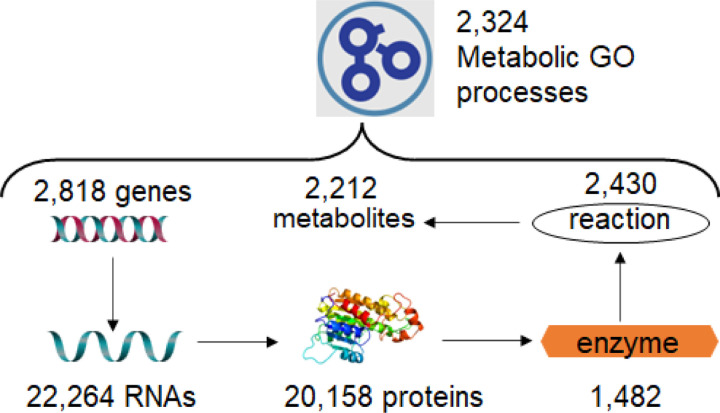
Content and relationships in the IDSL.GOA metabolic knowledgebase. Total number of metabolic GO terms under the metabolic process (GO:0008152) are 6,084. Of those, 2,546 had least one human gene annotated with and 2,324 had at least one metabolite linked with.

To perform IDSL.GOA over-representation analysis, we first needed to create a database of relationships among metabolic entities. This database was designed to capture the heterogenous relationships among genes, proteins, RNA nucleotides (transcripts), enzymes, reactions, compounds, and gene ontology terms. The source data for these relationships were obtained from various publicly available key databases, including the NCBI, Expasy – SIB Swiss Institute of Bioinformatics, KEGG, and the Gene Ontology Consortium (Table 1). We restricted the knowledgebase to only human genes and their products in the first version of the KB. The resulting version 1 of the IDSL.GOA database contained a total of 2,818 genes, 20,158 protein sequences, 22,264 RNA variants (transcripts), 1,482 enzyme commission numbers, 2,430 reactions, 2,212 compounds, and 2,324 gene ontology terms for metabolic processes (Figure 1).

The linkages among these entities had a power-law distribution. In comparison to the Reactome database^26^, IDSL.GOA KB had 210 more metabolites linked with metabolite pathways and processes. Overall, the IDSL.GOA database provided a comprehensive resource for performing GO over-representation analysis for metabolite lists.

### Aging mouse brain metabolomics- a case study:

**Figure 2:**
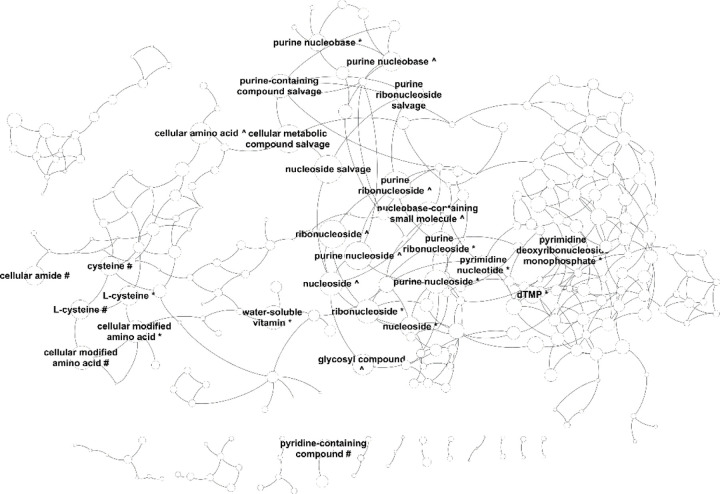
GO Tree visualization of the significantly overrepresented GO-terms in the input metabolite list. For clarity, only the top selected GO terms are labelled. Complete network is available in the Cytoscape session file in the SI material. # denotes – catabolic process, ^ denotes biosynthesis process and * denotes metabolic process.

**Figure 3.**
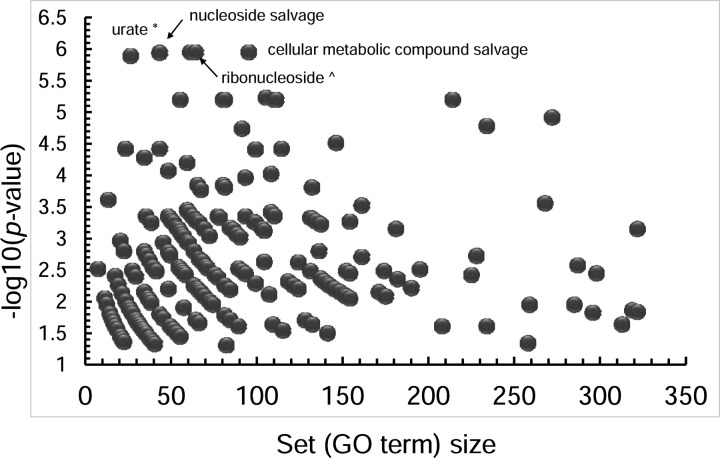
IDSL.GOA impact plot to show the most overrepresented GO terms by their specificity. A small set size shows more specific metabolic processes. For clarity, only the top metabolic processes are labelled but a fully labelled graphics is provided in the (Figure S1)

In this study, we aimed to investigate the changes in metabolite levels in the brain cortex of old and young mice using a metabolomics atlas that contained close to 1,547 identified compounds, out of which 389 were linked to KEGG compound identifiers. We identified 557 metabolites that were significantly different between the old (59 weeks) and young (3 weeks) female mouse brain cortex (Table S1). Out of those significant ones, 96 had KEGG identifiers available, which were used as input for IDSL.GOA analysis. The GO analysis results suggested a total of 282 GO processes that were overrepresented in the input list at an FDR cutoff of 0.05 (Table S2). The GO network and the impact plot visualization suggested that processes in nucleotide and amino acid metabolism (GO:0043174, GO:0046415 and GO:0006166) were significantly affected during the aging process (Figure 2–3, Table S2, Figure S1).

**Figure 4:**
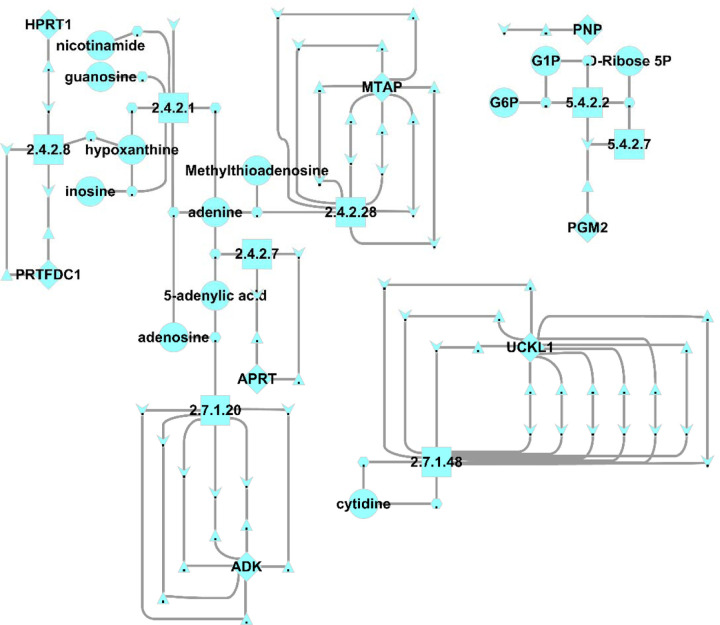
A focused biochemical network visualization for a nucleoside salvage GO process (GO:0043174). Only genes, EC numbers and compounds are labelled for clarity. ♦– gene, ▲ – RNA, **V** – protein, ■ – enzyme, - reaction, ● – compound.

Next, we used the reaction network visualization feature in IDSL.GOA to create a more accurate representation of the nucleotide salvage pathway (GO:0043174) (Figure 4 and Figure S2). This revealed key genes, Hypoxanthine-guanine phosphoribosyltransferase (HPRT1), methylthioadenosine phosphorylase(MTAP), Purine Nucleoside Phosphorylase (PNP) and Adenine phosphoribosyltransferase (APRT) in nucleotide salvage pathways and their enzymatic reactions which potentially were affected during the aging process.

### IDSL.GOA online tool:

The IDSL.GOA online tool is a user-friendly resource for identifying overrepresented metabolic processes in a list of metabolites. The online interface offers features including analysis, query, explore, statistic and download options. The ‘Run Go Analysis’ option on the landing page allows users to input a list of KEGG identifiers and obtain results in various formats, including Cytoscape SIF, Microsoft Excel, and CSV. The KEGG identifiers for only the significant compounds (p<0.05) in a statistical test should be used as input. The Cytoscape SIF and node attribute files are useful for creating high-resolution figures in the Cytoscape desktop software^29^. The primary analysis results are visualized in a ‘GO Ontology network’ graph using Cytoscape JS library, which provides an intuitive and interactive way to explore the data. This view is analogous to the pathway ontology visualization in the Reactome database^26^. The size of the node in the graph reflects the significance of the term, with larger nodes indicating more significant terms in the hypergeometric test. Additionally, an impact plot shows how specific the GO terms are for the input list, by plotting the set size versus −log(p-value). The explore option allows users to navigate the GO ontology tree and access GO-term-specific metabolic reaction network graphs. Clicking on a GO term in the main analysis, query or explore options provide the GO-term specific metabolic reaction network graph that contains genes, transcripts, proteins, enzymes, reactions and reactants. This reaction network feature is analogous to conventional pathway maps but here we create these maps automatically and have the flexibility to create new reaction layouts with the most comprehensive biochemical view a metabolic process (Figure 3). The reaction network for a GO term can be visualized for all the reactions or only the ones that were provided in the input file. The reaction network panel also allows querying a single GO-term by typing the GO id. Clicking on a molecular entity in the GO reaction network will get the NCBI and KEGG database hyperlinks that can be used for obtaining more information about the entity. The query option allows users to query a single compound, reaction, gene, protein and transcript to retrieve the associated metabolic GO terms. All GO network visualization has a basic set of layouts (views) implemented which can be explored by a user to find the most readable and helpful views for a GO ontology network and the reaction network that can aid in the biological interpretation of metabolite lists. Finally, the statistics and download tabs provide updates on the database version and download links, and the landing page offers Instructions for using the database. The GO ontology network visualizes the enrichment statistics and GO-specific reaction networks are two key and novel features for IDSL.GOA online tool, making it a valuable resource for the metabolomics community.

### Comparison with other pathway analysis tools:

To compare our IDSL.GOA results against existing approaches, we queried the same list of 96 significant KEGG identifiers against MetaboAnalyst and Reactome Pathway Analysis, two commonly used tools for metabolomics data interpretations. However, the results obtained from both tools were drastically different from those obtained using IDSL.GOA. First, on the FDR cutoff of 0.05, MetaboAnalyst identified only 4 pathways, and Reactome identified only 2 pathways. This result was likely caused by MetaboAnalyst’s use of a manually curated list of 80 pathways which may not concord with GO ontology, and which may have poor coverage for the input list. The most significant pathways for the input list were related to amino acid metabolism (Table S3). It also did not provide a one-to-one linking of metabolites to pathways, making it difficult to provide a coverage comparison between these tools. The Reactome pathways analysis could not map 48 compounds (50%) to any of 547 tested metabolic pathways (Table S4). In contrast, IDSL.GOA KB missed 34 compounds (35%). The poor coverage of compounds in the Reactome database may explain why only two pathways passed the FDR cutoff. We found that MetaboAnalyst and Reactome Pathway Analysis had limitations in terms of providing a comprehensive coverage of the metabolites linked to pathways, as well as in their ability to identify accurate pathways and processes that are related to the aging process. In comparison to these two tools, IDSL.GOA has several unique features - 1) GO database 2) focused reaction network 3) flexible and interactive data visualization 4) GO terms sorting by relevance and specificity in the impact plot 4) integrated with the GO ontology dataset and the NCBI database resources. Overall, the results of our study demonstrate that the use of IDSL.GOA can improve the mechanistic interpretation of metabolomics data, allowing for the identification of key biological processes involved in complex biological phenomena such as aging.

## Discussion:

IDSL.GOA is the first bioinformatics tool that used GO terms for over-representation analysis of metabolomics datasets. By mapping the metabolites to their associated GO terms, IDSL.GOA can improve the mechanistic interpretation of metabolomics data by providing a functional annotation of the metabolites based on their associated metabolic processes and pathways in the Gene Ontology database. It is a more sensitive and accurate tool for data with larger lists (>1000 named metabolites)^8, 9^. This can lead to the identification of key regulatory pathways and molecular mechanisms that are involved in the observed changes and can guide further experimentation and hypothesis testing. By leveraging the new IDSL.GOA knowledgebase, we were able to identify the overrepresented metabolic pathways and processes in our caste study dataset and gain new insights into the underlying mechanisms that govern metabolic activity in aging brain tissue.

### Advantages of using GO terms for metabolomics data interpretation:

There are several advantages of GO analysis over traditional pathway analysis. GO analysis provides a more comprehensive annotation system for genes and their products than pathway analysis, allowing for a broader range of metabolic processes and pathways to be analyzed^30^. Unlike pathway analysis, a GO analysis is not limited to hand-drawn pathway maps which tend to differ from one database to another, making it more flexible and adaptable to different experimental conditions. Depending on the background pathway database, the interpretation of metabolite lists can differ and may be inaccurate, leading to contradicting results and less impact^10^. On contrast, GO analysis allows for a more detailed and accurate interpretation of results, as it provides a broader context for the function and regulation of metabolite levels. Because the GO system is standardized, it allows for greater consistency and comparability between different studies and datasets. GO terms not only covers the known pathway maps but also covers additional metabolic processes that are not yet included in the pathway databases.

### Metabolic reaction network for GO terms:

An innovative feature of IDSL.GOA is to create focused reaction network for metabolic GO terms that combines genes, transcripts, proteins, and reactions in one view. The feature enables creating pathway maps like diagrams for GO-terms in the Cytoscape software. By providing a clear and intuitive visual representation of the metabolic pathway, the focus reaction network visualization can facilitate data interpretation and hypothesis generation, and ultimately lead to a better understanding of metabolic function and regulation. The focused network used the atomic mapping of reactant pairs to create the graph, which provides a more accurate view of the metabolic reactions^22^. The partial network visualization subset the GO term specific reaction network for only the compounds that were detected in the specific study or specimen being analyzed. This view can be useful for multi-omics integration as it shows genes, transcripts, proteins and metabolites all in one connected view.

### Key strengths of IDSL.GOA tool:

The IDSL.GOA tool is a free, user-friendly and web-based platform that utilizes Gene Ontology (GO) terms for the analysis of metabolomics data. It offers an intuitive interface that allows users to perform GO enrichment analysis for an input metabolite list. The tool has a range of useful features to facilitate the interpretation and has a wide range of capabilities, including query, explore, statistics, and download options. Additionally, IDSL.GOA offers a focused reaction network visualization for an in-depth mechanistic interpretation of metabolomics data. The use of GO terms provides an improved biological interpretation of metabolomics data, which can help researchers identify novel and metabolically relevant pathways and processes. The tool is built on a robust knowledgebase that contains relationships among metabolic entities, obtained from various sources including NCBI, Expasy, KEGG, and the Gene Ontology Consortium databases. The tool allows for a more comprehensive and accurate analysis of metabolomics data by identifying not only the predefined pathways but also relevant metabolic processes that are not included in the commonly used pathway databases. It is the first of its kind tool for metabolomics data.

### IDSL.GOA and multi-omics integration:

GO analysis can be used to integrate different types of data, such as genomics, transcriptomics, proteomics, and metabolomics data, providing a more complete metabolic view of biological systems^30^. IDSL.GOA can be used for multi-omics integration by combining the results of gene expression analysis and metabolite profiling. This can be achieved by comparing the GO term enrichment results obtained from the separate gene expression and metabolite profiling analyses and identifying the common significantly enriched GO terms. This integration has not been achieved before IDSL.GOA since there was not a single knowledgebase developed that created the GO-term specific focused reaction networks. IDSL.GOA can also support the statistical multi-omics analysis to extract meaningful biological information from correlated features across multiple omics datasets^31^. By integrating the results of IDSL.GOA with the results of statistical multi-omics analysis, researchers can obtain a more comprehensive understanding of the underlying biological mechanisms that are involved in the disease or condition of interest. IDSL.GOA’s unique feature of creating a comprehensive reaction network with genes, transcripts, proteins, and metabolites all in one view, along with its ability to subset the view based on detected compounds, make it a powerful tool for multi-omics integration and reaction network visualization.

### Limitations:

Few limitations should be noted. The IDSL.GOA tool relies on the availability of KEGG-linked metabolite data, and the coverage of metabolite curation may vary across different metabolomics laboratories. Not all KEGG compound identifiers are linked to reactions and enzyme commission numbers. The GO hierarchy and associated annotations may contain biases or inaccuracies due to incomplete or outdated information. There is some redundancy in GO term names which may inflate the over-representation analysis results. The mechanistic interpretation still needs to be validated by additional experimentation. By discussing these limitations, we can provide a more balanced view of the capabilities and potential drawbacks of the IDSL.GOA tool for GO analysis in metabolomics.

## Conclusions:

In summary, the IDSL.GOA tool can enable a comprehensive and accurate biological interpretation of metabolomics data. A much-needed transition from pathway maps to GO terms for interpreting metabolomics datasets can be supported by the IDSL.GOA tool. It is more sensitive in identifying significantly enriched GO terms that are relevant for metabolic processes. It also provides a powerful and user-friendly approach for integrating multi-omics data and identifying the biological pathways and processes. By providing a comprehensive view of the underlying biology, this approach can facilitate the identification of key regulatory pathways and biomarkers that may be useful for diagnosis, prognosis, and therapeutic targeting.

## Supplementary Material

Supplement 1 Table S1 : Significantly different metabolites between the older vs younger brain cortex region.
Table S2 : Full results for the Gene Ontology Analysis

Supplement 2 Figure S1: IDSL.GOA impact plot with all labels

Supplement 3 Figure S2: Nucleotide salvage GO metabolic process with all the molecular entities

Supplement 4 File S1: Gene Ontology Network for the over-represented metabolic processes.

## Figures and Tables

**Table 1:** Data sources for assembling the IDSL.GOA knowledgebase.

Resource	Address	Information
Gene Ontology	http://purl.obolibrary.org/obo/go.obo	GO-GO
Expasy Enzyme	https://ftp.expasy.org/databases/enzyme/enzyme.dat	EC Numbers
NCBI Gene Accession	https://ftp.ncbi.nlm.nih.gov/gene/DATA/gene2accession.gz	Gene – Transcript Transcript - Protein
NCBI Gene GO Annotation	https://ftp.ncbi.nlm.nih.gov/gene/DATA/gene2go.gz	Gene - GO
NCBI Eutils API	https://eutils.ncbi.nlm.nih.gov/entrez/eutils/esearch.fcgi	EC-protein
KEGG REST API	http://rest.kegg.jp	EC-Reaction Reaction–Metabolite

Note: These information were accessed in January 2023.

## References

[1] Brunk E.; Sahoo S.; Zielinski D. C.; Altunkaya A.; Drager A.; Mih N.; Gatto F.; Nilsson A.; Preciat Gonzalez G. A.; Aurich M. K.; Recon3D enables a three-dimensional view of gene variation in human metabolism. Nat Biotechnol 2018, 36 (3), 272–281. DOI: 10.1038/nbt.4072 29457794 PMC5840010

[2] Romero P.; Wagg J.; Green M. L.; Kaiser D.; Krummenacker M.; Karp P. D. Computational prediction of human metabolic pathways from the complete human genome. Genome Biol 2005, 6 (1), R2. DOI: 10.1186/gb-2004-6-1-r2 15642094 PMC549063

[3] Horgusluoglu E.; Neff R.; Song W. M.; Wang M.; Wang Q.; Arnold M.; Krumsiek J.; Galindo-Prieto B.; Ming C.; Nho K.; Integrative metabolomics-genomics approach reveals key metabolic pathways and regulators of Alzheimer’s disease. Alzheimers Dement 2022, 18 (6), 1260–1278. DOI: 10.1002/alz.12468 34757660 PMC9085975

[4] Chen Y.; Lu T.; Pettersson-Kymmer U.; Stewart I. D.; Butler-Laporte G.; Nakanishi T.; Cerani A.; Liang K. Y. H.; Yoshiji S.; Willett J. D. S.; Genomic atlas of the plasma metabolome prioritizes metabolites implicated in human diseases. Nat Genet 2023, 55 (1), 44–53. DOI: 10.1038/s41588-022-01270-1 36635386 PMC7614162

[5] Goodrich J. A.; Walker D. I.; He J.; Lin X.; Baumert B. O.; Hu X.; Alderete T. L.; Chen Z.; Valvi D.; Fuentes Z. C.; Metabolic Signatures of Youth Exposure to Mixtures of Per- and Polyfluoroalkyl Substances: A Multi-Cohort Study. Environ Health Perspect 2023, 131 (2), 27005. DOI: 10.1289/EHP11372 36821578 PMC9945578

[6] Pi H.; Xia L.; Ralph D. D.; Rayner S. G.; Shojaie A.; Leary P. J.; Gharib S. A. Metabolomic Signatures Associated With Pulmonary Arterial Hypertension Outcomes. Circ Res 2023, 132 (3), 254–266. DOI: 10.1161/CIRCRESAHA.122.321923 36597887 PMC9904878

[7] Witkowski M.; Nemet I.; Alamri H.; Wilcox J.; Gupta N.; Nimer N.; Haghikia A.; Li X. S.; Wu Y.; Saha P. P.; The artificial sweetener erythritol and cardiovascular event risk. Nat Med 2023. DOI: 10.1038/s41591-023-02223-9 PMC10334259 36849732

[8] Ding J.; Ji J.; Rabow Z.; Shen T.; Folz J.; Brydges C. R.; Fan S.; Lu X.; Mehta S.; Showalter M. R.; A metabolome atlas of the aging mouse brain. Nat Commun 2021, 12 (1), 6021. DOI: 10.1038/s41467-021-26310-y 34654818 PMC8519999

[9] Byeon S. K.; Madugundu A. K.; Garapati K.; Ramarajan M. G.; Saraswat M.; Kumar M. P.; Hughes T.; Shah R.; Patnaik M. M.; Chia N.; Development of a multiomics model for identification of predictive biomarkers for COVID-19 severity: a retrospective cohort study. Lancet Digit Health 2022, 4 (9), e632–e645. DOI: 10.1016/S2589-7500(22)00112-1 35835712 PMC9273185

[10] Wieder C.; Frainay C.; Poupin N.; Rodriguez-Mier P.; Vinson F.; Cooke J.; Lai R. P.; Bundy J. G.; Jourdan F.; Ebbels T. Pathway analysis in metabolomics: Recommendations for the use of over-representation analysis. PLoS Comput Biol 2021, 17 (9), e1009105. DOI: 10.1371/journal.pcbi.1009105 34492007 PMC8448349

[11] Sarkar A.; Jin Y.; DeFelice B. C.; Logan C. Y.; Yang Y.; Anbarchian T.; Wu P.; Morri M.; Neff N. F.; Nguyen H.; Intermittent fasting induces rapid hepatocyte proliferation to restore the hepatostat in the mouse liver. Elife 2023, 12. DOI: 10.7554/eLife.82311 PMC9889086 36719070

[12] Tanes C.; Bittinger K.; Gao Y.; Friedman E. S.; Nessel L.; Paladhi U. R.; Chau L.; Panfen E.; Fischbach M. A.; Braun J.; Role of dietary fiber in the recovery of the human gut microbiome and its metabolome. Cell Host Microbe 2021, 29 (3), 394–407 e395. DOI: 10.1016/j.chom.2020.12.012 33440171 PMC8022197

[13] Hunt N. J.; Kang S. W. S.; Lockwood G. P.; Le Couteur D. G.; Cogger V. C. Hallmarks of Aging in the Liver. Comput Struct Biotechnol J 2019, 17, 1151–1161. DOI: 10.1016/j.csbj.2019.07.021 31462971 PMC6709368

[14] Yuan J. M.; Gao Y. T.; Murphy S. E.; Carmella S. G.; Wang R.; Zhong Y.; Moy K. A.; Davis A. B.; Tao L.; Chen M.; Urinary levels of cigarette smoke constituent metabolites are prospectively associated with lung cancer development in smokers. Cancer Res 2011, 71 (21), 6749–6757. DOI: 10.1158/0008-5472.CAN-11-0209 22028322 PMC3392910

[15] Surendran P.; Stewart I. D.; Au Yeung V. P. W.; Pietzner M.; Raffler J.; Worheide M. A.; Li C.; Smith R. F.; Wittemans L. B. L.; Bomba L.; Rare and common genetic determinants of metabolic individuality and their effects on human health. Nat Med 2022, 28 (11), 2321–2332. DOI: 10.1038/s41591-022-02046-0 36357675 PMC9671801

[16] Vasilopoulou C. G.; Sulek K.; Brunner A. D.; Meitei N. S.; Schweiger-Hufnagel U.; Meyer S. W.; Barsch A.; Mann M.; Meier F. Trapped ion mobility spectrometry and PASEF enable in-depth lipidomics from minimal sample amounts. Nat Commun 2020, 11 (1), 331. DOI: 10.1038/s41467-019-14044-x 31949144 PMC6965134

[17] Koopman J.; Grimme S. From QCEIMS to QCxMS: A Tool to Routinely Calculate CID Mass Spectra Using Molecular Dynamics. J Am Soc Mass Spectrom 2021, 32 (7), 1735–1751. DOI: 10.1021/jasms.1c00098 34080847

[18] Barupal D. K.; Fan S.; Fiehn O. Integrating bioinformatics approaches for a comprehensive interpretation of metabolomics datasets. Curr Opin Biotechnol 2018, 54, 1–9. DOI: 10.1016/j.copbio.2018.01.010 29413745 PMC6358024

[19] Bonini P.; Kind T.; Tsugawa H.; Barupal D. K.; Fiehn O. Retip: Retention Time Prediction for Compound Annotation in Untargeted Metabolomics. Anal Chem 2020, 92 (11), 7515–7522. DOI: 10.1021/acs.analchem.9b05765 32390414 PMC8715951

[20] Lind L.; Fall T.; Arnlov J.; Elmstahl S.; Sundstrom J. Large-Scale Metabolomics and the Incidence of Cardiovascular Disease. J Am Heart Assoc 2023, 12 (2), e026885. DOI: 10.1161/JAHA.122.026885 36645074 PMC9939066

[21] Barupal D. K.; Fiehn O. Chemical Similarity Enrichment Analysis (ChemRICH) as alternative to biochemical pathway mapping for metabolomic datasets. Sci Rep 2017, 7 (1), 14567. DOI: 10.1038/s41598-017-15231-w 29109515 PMC5673929

[22] Barupal D. K.; Haldiya P. K.; Wohlgemuth G.; Kind T.; Kothari S. L.; Pinkerton K. E.; Fiehn O. MetaMapp: mapping and visualizing metabolomic data by integrating information from biochemical pathways and chemical and mass spectral similarity. BMC Bioinformatics 2012, 13, 99. DOI: 10.1186/1471-2105-13-99 22591066 PMC3495401

[23] Barupal D. K.; Fiehn O. Generating the Blood Exposome Database Using a Comprehensive Text Mining and Database Fusion Approach. Environ Health Perspect 2019, 127 (9), 97008. DOI: 10.1289/EHP4713 31557052 PMC6794490

[24] Kanehisa M.; Furumichi M.; Sato Y.; Kawashima M.; Ishiguro-Watanabe M. KEGG for taxonomy-based analysis of pathways and genomes. Nucleic Acids Res 2023, 51 (D1), D587–D592. DOI: 10.1093/nar/gkac963 36300620 PMC9825424

[25] Karp P. D.; Billington R.; Caspi R.; Fulcher C. A.; Latendresse M.; Kothari A.; Keseler I. M.; Krummenacker M.; Midford P. E.; Ong Q.; The BioCyc collection of microbial genomes and metabolic pathways. Brief Bioinform 2019, 20 (4), 1085–1093. DOI: 10.1093/bib/bbx085 29447345 PMC6781571

[26] Gillespie M.; Jassal B.; Stephan R.; Milacic M.; Rothfels K.; Senff-Ribeiro A.; Griss J.; Sevilla C.; Matthews L.; Gong C.; The reactome pathway knowledgebase 2022. Nucleic Acids Res 2022, 50 (D1), D687–D692. DOI: 10.1093/nar/gkab1028 34788843 PMC8689983

[27] Gene Ontology C. The Gene Ontology resource: enriching a GOld mine. Nucleic Acids Res 2021, 49 (D1), D325–D334. DOI: 10.1093/nar/gkaa1113 33290552 PMC7779012

[28] Pang Z.; Zhou G.; Ewald J.; Chang L.; Hacariz O.; Basu N.; Xia J. Using MetaboAnalyst 5.0 for LC-HRMS spectra processing, multi-omics integration and covariate adjustment of global metabolomics data. Nat Protoc 2022, 17 (8), 1735–1761. DOI: 10.1038/s41596-022-00710-w 35715522

[29] Shannon P.; Markiel A.; Ozier O.; Baliga N. S.; Wang J. T.; Ramage D.; Amin N.; Schwikowski B.; Ideker T. Cytoscape: a software environment for integrated models of biomolecular interaction networks. Genome Res 2003, 13 (11), 2498–2504. DOI: 10.1101/gr.1239303 14597658 PMC403769

[30] Gu Y.; Zhou Y.; Ju S.; Liu X.; Zhang Z.; Guo J.; Gao J.; Zang J.; Sun H.; Chen Q.; Multi-omics profiling visualizes dynamics of cardiac development and functions. Cell Rep 2022, 41 (13), 111891. DOI: 10.1016/j.celrep.2022.111891 36577384

[31] Maitre L.; Bustamante M.; Hernandez-Ferrer C.; Thiel D.; Lau C. E.; Siskos A. P.; Vives-Usano M.; Ruiz-Arenas C.; Pelegri-Siso D.; Robinson O.; Multi-omics signatures of the human early life exposome. Nat Commun 2022, 13 (1), 7024. DOI: 10.1038/s41467-022-34422-2 36411288 PMC9678903

