# Supplementary material for: IDSL.GOA: Gene Ontology Analysis for Metabolomics
Source: bioRxiv. 2023 Jul 3:2023.03.25.534225. Preprint. [Version 3] doi: 10.1101/2023.03.25.534225 (PMC10081191; doi:10.1101/2023.03.25.534225)
Supplement: Supplement 2 — Figure S1: IDSL.GOA impact plot with all labels [file media-2.pptx]

## Slide 1
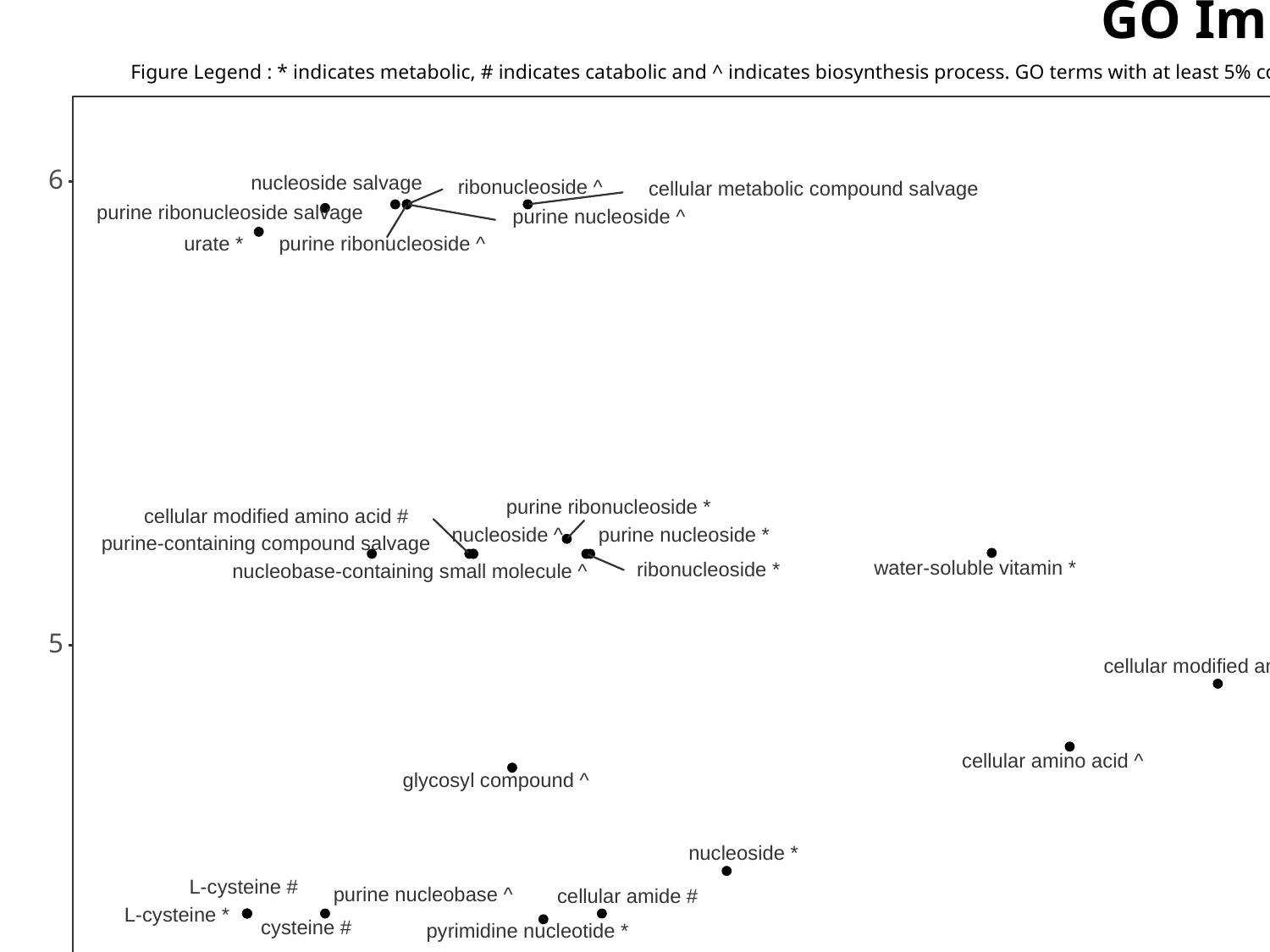

GO Impact Plot
Figure Legend : * indicates metabolic, # indicates catabolic and ^ indicates biosynthesis process. GO terms with at least 5% coverage are shown in this plot.
6
nucleoside salvage
cellular metabolic compound salvage
ribonucleoside ^
purine ribonucleoside salvage
purine nucleoside ^
purine ribonucleoside ^
urate *
purine ribonucleoside *
cellular modified amino acid #
purine nucleoside *
nucleoside ^
purine-containing compound salvage
water-soluble vitamin *
nucleobase-containing small molecule ^
ribonucleoside *
5
cellular modified amino acid *
cellular amino acid ^
glycosyl compound ^
nucleoside *
L-cysteine #
purine nucleobase ^
cellular amide #
L-cysteine *
cysteine #
pyrimidine nucleotide *
pyridine-containing compound #
pyrimidine deoxyribonucleoside monophosphate *
dTMP *
purine nucleobase *
4
deoxyribonucleoside monophosphate *
pyrimidine deoxyribonucleotide *
nucleobase-containing small molecule #
nucleoside #
pyrimidine nucleoside monophosphate *
pyrimidine-containing compound *
purine ribonucleoside monophosphate *
IMP #
ribonucleoside #
glycerol ^
nucleobase ^
purine nucleoside monophosphate *
alpha-amino acid ^
pyrimidine nucleoside monophosphate #
-log (pvalue)
allantoin *
alditol ^
purine deoxyribonucleoside monophosphate #
glycosyl compound #
nucleotide salvage
deoxyribonucleoside monophosphate #
AMP *
organophosphate #
aspartate family amino acid *
dAMP #
purine ribonucleoside monophosphate ^
deoxyribonucleotide *
purine deoxyribonucleoside monophosphate *
sulfur amino acid *
sulfur amino acid #
deoxyribonucleotide #
nucleobase *
AMP ^
nucleoside monophosphate *
dAMP *
IMP *
2'-deoxyribonucleotide *
dTMP #
ribonucleoside monophosphate *
deoxyribose phosphate *
purine deoxyribonucleotide *
nucleoside monophosphate #
pyrimidine deoxyribonucleotide #
purine nucleoside monophosphate #
dCMP #
ribonucleoside monophosphate #
deoxyribose phosphate #
pyridine-containing compound *
purine ribonucleoside monophosphate #
dGMP #
ribonucleoside monophosphate ^
nucleoside monophosphate ^
dUMP #
adenosine *
AMP #
dGMP *
thiamine-containing compound *
glycosyl compound *
aspartate family amino acid ^
hypoxanthine ^
thiamine *
sulfur compound *
purine nucleoside monophosphate ^
dCMP *
pyrimidine ribonucleoside monophosphate #
purine nucleobase salvage
GMP *
deoxyinosine #
3
cysteine *
vitamin *
GMP ^
deoxyadenosine #
UMP #
ribonucleoside diphosphate #
hexose ^
adenosine ^
glutamine family amino acid *
pyrimidine nucleotide #
dephosphorylation
purine deoxyribonucleoside #
heterocycle ^
GMP salvage
ADP #
carbohydrate ^
hypoxanthine *
monosaccharide ^
inosine #
dUMP *
inosine *
purine ribonucleotide salvage
pyrimidine ribonucleotide *
pyrimidine nucleoside *
purine deoxyribonucleoside *
purine ribonucleoside #
nucleoside phosphate #
deoxyinosine *
UMP *
pyrimidine nucleotide ^
ribonucleoside triphosphate *
glutamine family amino acid #
phosphorylation
AMP salvage
purine ribonucleotide #
choline *
organophosphate ^
cellular modified amino acid ^
pyrimidine ribonucleotide #
deoxyadenosine *
alditol *
L-methionine salvage
gamma-aminobutyric acid *
nucleobase-containing compound #
ribonucleotide #
purine nucleoside #
cellular carbohydrate ^
deoxyribonucleoside monophosphate ^
proteolysis
inositol ^
dicarboxylic acid *
taurine *
ammonium ion *
glutamate *
amino acid salvage
purine-containing compound ^
purine nucleotide #
glycerol *
ADP *
glutamate #
alkanesulfonate *
nucleotide phosphorylation
sulfur amino acid ^
L-methionine ^
aspartate family amino acid #
purine ribonucleoside diphosphate #
deoxyribonucleoside #
ATP *
nucleobase-containing compound ^
purine ribonucleoside triphosphate *
purine ribonucleoside diphosphate *
alkanesulfonate ^
purine nucleoside diphosphate #
dicarboxylic acid #
purine nucleoside diphosphate *
polyol ^
folic acid *
S-adenosylmethionine *
ribonucleoside diphosphate *
purine nucleoside triphosphate *
deoxyribonucleoside *
gluconeogenesis
taurine ^
aldehyde #
nucleotide #
pteridine-containing compound *
nucleoside diphosphate *
purine nucleotide salvage
mRNA *
peptidyl-lysine deacetylation
carbohydrate derivative #
purine-containing compound #
nucleoside triphosphate *
histone deacetylation
protein deacetylation
one-carbon *
serine family amino acid *
gamma-aminobutyric acid ^
2
L-serine ^
DNA *
IMP ^
neurotransmitter ^
cellular aldehyde *
L-alanine #
ATP generation from poly-ADP-D-ribose
glycerophospholipid #
glycine ^, by transamination of glyoxylate
folic acid-containing compound *
L-lysine # to acetyl-CoA via saccharopine
polyol *
nucleotide ^
L-alanine *
macromolecule deacylation
xanthine *
adenosine #
histidine #
protein deacylation
deoxyribonucleoside triphosphate #
L-serine *
ornithine *
nucleoside phosphate ^
phospholipid #
purine nucleoside triphosphate #
amine #
diadenosine polyphosphate *
DNA protection
adenine *
mRNA #
methionine *
cellular carbohydrate *
cellular biogenic amine #
glyoxylate #
serine family amino acid ^
RNA #
glutathione ^
L-lysine # to acetyl-CoA
neurotransmitter *
RNA *
hexose *
L-lysine *
IMP salvage
glycerophospholipid *
sulfur compound #
purine deoxyribonucleoside triphosphate *
glycerolipid #
glycine betaine ^
glycine betaine *
arginine *
peptide ^
cysteine ^
glycine betaine ^ from choline
L-lysine #
histidine *
inositol *
glucose *
nonribosomal peptide ^
aspartate #
proline ^
choline #
methionine ^
lysine #
polyol #
lysine *
L-proline ^
2-oxoglutarate *
ribose phosphate ^
pyrimidine nucleoside #
hydrogen sulfide *
polyamine ^
zymogen activation
proline *
triglyceride #
4-hydroxyproline *
glutathione #
cellular biogenic amine ^
polyamine *
L-serine #
4-hydroxyproline #
0
200
400
600
GO Set Size
