# Supplementary figures and images for: IDSL.GOA: Gene Ontology Analysis for Metabolomics
Source: bioRxiv. 2023 Jul 3:2023.03.25.534225. Preprint. [Version 3] doi: 10.1101/2023.03.25.534225 (PMC10081191; doi:10.1101/2023.03.25.534225)

## Slide 1
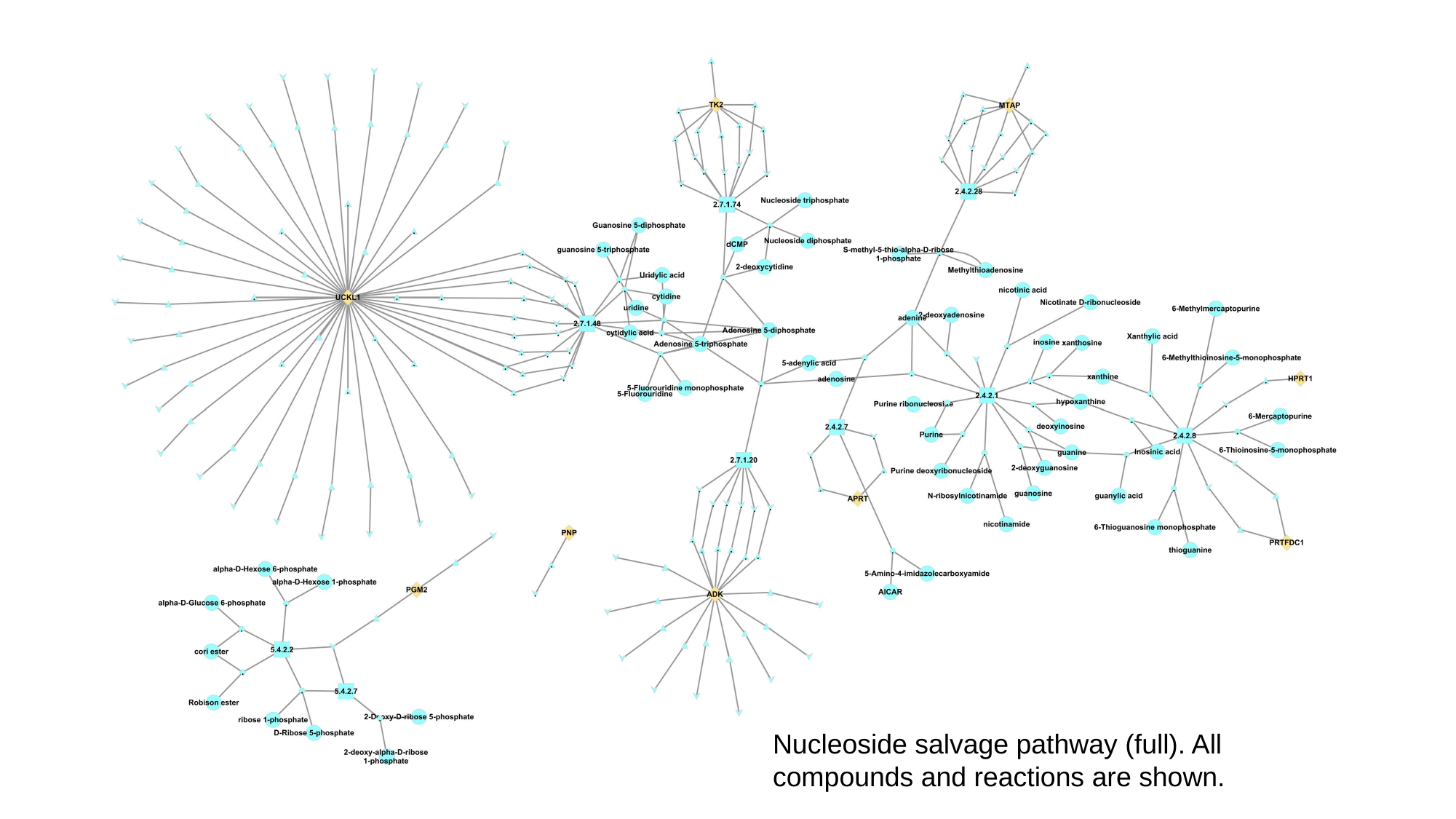

Nucleoside salvage pathway (full). All compounds and reactions are shown.

Supplement: Supplement 3 — Figure S2: Nucleotide salvage GO metabolic process with all the molecular entities [file media-3.pptx]
